# Twisting of a Pristine α-Fe Nanowire: From Wild Dislocation Avalanches to Mild Local Amorphization

**DOI:** 10.3390/nano11061602

**Published:** 2021-06-18

**Authors:** Yang Yang, Xiangdong Ding, Jun Sun, Ekhard K. H. Salje

**Affiliations:** 1 State Key Laboratory for Mechanical Behaviour of Materials, School of Materials Science and Engineering, Xi’an Jiaotong University, Xi’an 710049, China; yangymse@xjtu.edu.cn (Y.Y.); junsun@mail.xjtu.edu.cn (J.S.); 2 Department of Earth Sciences, University of Cambridge, Cambridge CB2 3EQ, UK

**Keywords:** α-Fe nanowire, torsion, dislocation avalanche, local amorphization, mild wild fluctuations, plasticity dynamics

## Abstract

The torsion of pristine α-Fe nanowires was studied by molecular dynamics simulations. Torsion-induced plastic deformation in pristine nanowires is divided into two regimes. Under weak torsion, plastic deformation leads to dislocation nucleation and propagation. Twisting-induced dislocations are mainly $\frac{1}{2}$<111> screw dislocations in a <112>-oriented nanowire. The nucleation and propagation of these dislocations were found to form avalanches which generate the emission of energy jerks. Their probability distribution function (PDF) showed power laws with mixing between different energy exponents. The mixing stemmed from simultaneous axial and radial dislocation movements. The power-law distribution indicated strongly correlated ‘wild’ dislocation dynamics. At the end of this regime, the dislocation pattern was frozen, and further twisting of the nanowire did not change the dislocation pattern. Instead, it induced local amorphization at the grip points at the ends of the sample. This “melting” generated highly dampened, mild avalanches. We compared the deformation mechanisms of twinned and pristine α-Fe nanowires under torsion.

## 1. Introduction

Metallic nanowires show high pseudoelasticity with full shape recovery from a uniaxial tensile strain larger than 40% [1,2,3,4]. Such an excellent shape recoverability in metallic nanowires defines their potential for applications as sensors, actuators, energy storage and transfer devices etc. in micro (nano)-electrical-mechanical systems (MEMS or NEMS). The driving force for pseudoelasticity in metallic nanowires under uniaxial loading is different from normal shape memory alloys [5]. There is no martensitic phase transformation in metallic nanowires under tension or compression. Instead, deformation and shape recovery are both realized by the movement of twin boundaries (TBs). The surface energy difference between the twinned and un-twinned regions is the driving force for pseudoelasticity. The energy barrier of TB motions in bcc metals such as Mo, W and Fe etc. is very small, therefore the energy dissipation is small. Nanosprings have been designed as a new energy storage device with a high efficiency of 98% based on body-centered cubic or bcc nanowires [1]. However, the driving by surface energy decreases rapidly with the increase of the wire size. Surface energy driven pseudoelasticity under tension is limited in metallic nanowires to diameters smaller than 5 nm [2]. Nevertheless, the synthesis of metallic nanowires thinner than 5 nm is still a challenge. In addition, a pure uniaxial stress field is difficult to achieve in applications. The applications of gradient stress fields such as bending and torsion are more common [6,7,8].

Bending or torsion-related pseudoelasticity also exists in metallic nanowires, where the main driving force comes from TBs (or interfaces) [9,10,11,12]. TBs show two different morphologies under bending or torsion. For example, the straight {112} TBs are curved after bending with accumulation of $\frac{1}{6}$<111> partial dislocations [10]. The repulsive force between dislocations results in a shape recovery during unloading. Normal <112> TBs can also transform into {110} interfaces possessing high interface energy in bending or torsion [9,11]. The high energy state is unstable when the external stress field decreases during unloading leading to shape recovery. Samples seeded with TBs show increased shape recoverability in nanowires with a diameter larger than 5 nm, because the existence of TBs delays the nucleation of dislocations. The dynamic deformation mechanism of TBs under torsion was studied by the statistical analysis of their induced nanostructure in our earlier work [11].

Besides twinning, dislocation slip [13] and stress-induced amorphization [14,15] are common plastic deformation modes in nanomaterials. Dislocations nucleate easier than twins when an α-Fe nanowire is thicker than 5 nm. The dislocation avalanche dynamics have been studied in bulk materials since the 1930s [16]. Jerky and intermittent dislocation-mediated plasticity have been observed in many hexagonal-closest-packed or hcp structures [17,18,19]. The jerks are correlated in space and time and are power-law distributed in size and energy. In face-centered cubic or fcc structures, wild and mild plasticity can coexist [20,21]. The wildness of the plasticity is strongly related to the sample size (e.g., “smaller is wilder”), to the interaction between dislocations, and to the interaction between dislocation and precipitations or defect clusters. Correlations between the wildness of dislocation avalanches and changes of the mean free path of dislocation movement was discussed in [21]. Dislocation movement can also be affected by the geometry of the sample. Typical thin nanowires have diameters of several tens of nanometers while the wires are very long. It is not known how dislocations move in such wires and what the avalanche dynamics [21] entails. In addition, the dynamic behavior of the stress-induced amorphization is unknown. We will address these questions in this paper.

## 2. Materials and Methods

**Figure 1:**
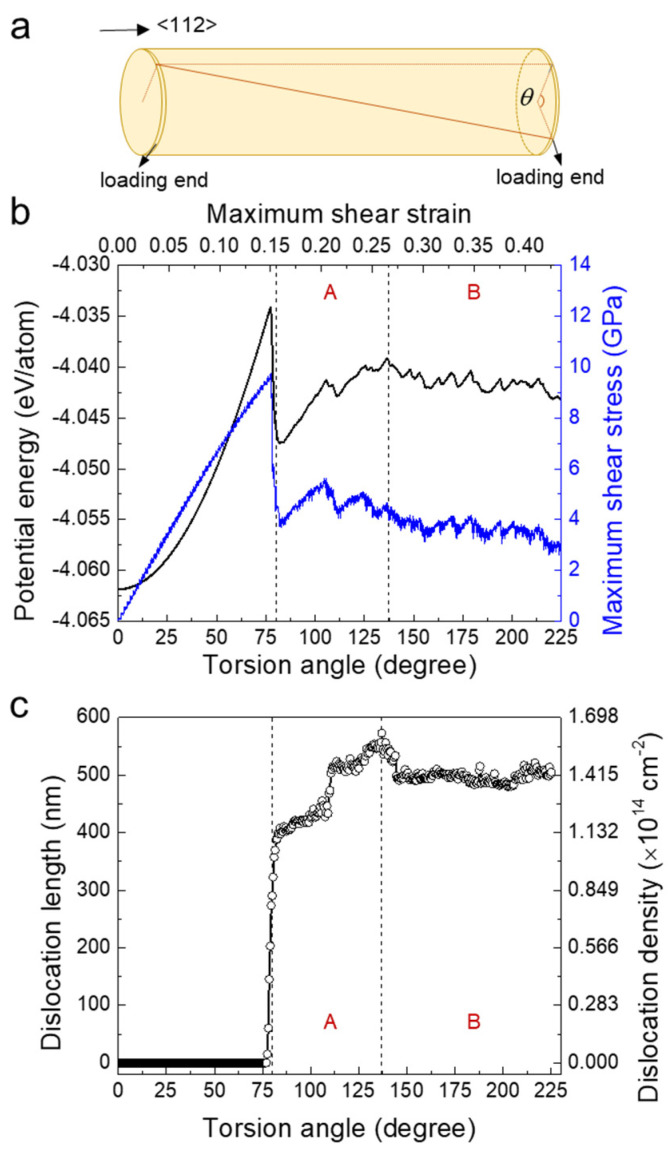
Torsion deformation of a pristine α-Fe nanowire with diameter of 10 nm and length of 45 nm at 1 K. (**a**). Schematic illustration of the twisting nanowire. (**b**) Evolution of maximum shear stress *τ*_max_ (blue) and mean potential energy *E*_p_ (black) with increase of torsion angle *θ* (maximum shear strain *γ*_max_). (**c**). Evolution of the total dislocation length and the dislocation density with the torsion angle *θ*.

We constructed a <112>-oriented α-Fe nanowire (Figure 1a) with a diameter of 10 nm and a length of 45 nm, at which size the surface effect can be neglected [2]. The nanowire had a circular cross section. There were ~300,000 atoms in this model. The interatomic potentials of α-Fe is described by Mendelev [22] using an embedded atom method (EAM) [23]. In the EAM potential, the interatomic energy is divided into two parts: first is the pair-wise interaction and second is the embedded energy from the contribution of electron charge density. The potential reproduces well several properties of α-Fe such as the elastic constants, interstitial and vacancy formation energy, and the six-fold core structure of screw dislocations [9]. The model was first relaxed at 1 K for 0.5 ns using a Nosé–Hoover thermostat [24,25]. After the relaxation, the nanowire was twisted by rotation of the loading ends, which contained 3 rigidly fixed atomic layers at each end of the wire. One loading end was fixed while the other was rotated around the axis of the nanowire. The torsion deformation was simulated at 1 K. The low temperature was chosen because thermal fluctuations can be ignored for the statistical analysis of the plastic deformation. Rotation was applied with a rate of 0.05° per picosecond, corresponding to a shear strain-rate of around 1 × 10^7^ s^−1^. We calculated the maximum shear strain *γ*_max_ as half of the torsion angle *θ* divided by the aspect ratio of the sample, *γ*_max_ = *θd*/(2*L*). The maximum shear stress was *τ*_max_ = *Td*/(2*I*_t_), where *T* was the torque applied and *I*_t_ was the polar moment of inertia. The molecular dynamics (MD) calculations were carried out in a canonical ensemble (NVT) using the Large-scale Atomic/Molecular Massively Parallel Simulator (LAMMPS) code [26] (version 29 Oct, 2020, USA). The atomic configurations are displayed by AtomEye [27] (version 3, 2012, USA). The type and configurations of dislocations generated during deformation was detected using the OVITO code [28,29] (version 3.3.5, 2020, Germany). The atomic volume was computed by the Voro++ package [30] (version 0.4.6, 2013, USA).

We use the statistical behavior of jerks initiated by torsion to analyze the dynamics of the twisting plasticity in a pristine α-Fe nanowire. A jerk is an energy discontinuity caused by structural changes (such as dislocation nucleation and movement, local melting etc.) Jerks are quantified by their strength *J*, which can be calculated as the square of the first derivation of the potential energy *E*_p_ with respect to time *t*. This quantity is known as a slew rate in electrical engineering, plasma physics etc. [31]. Our previous work showed that there is no difference in the statistical behavior between the jerk strength and other parameters such as the jerk energy [11,32]. The probability distribution function (PDF) is used to describe the plasticity dynamics. A power-law PDF indicates strong collaborative movements, which indicates “wild” plasticity. Power-law distributed avalanches are scale invariant over large energy intervals [33]. In contrast, an exponential PDF indicates that the movements are random, which is coined “mild” [11,20]. These movements are not scale invariant.

## 3. Results

### 3.1. Two-Stage Plasticity in a Twisted Pristine α-Fe Nanowire

Figure 1b shows the evolution of maximum shear stress (blue) as a function of the torsion angle *θ* (maximum shear strain *γ*_max_) at 1 K for a pristine α-Fe nanowire with a diameter of 10 nm and a length of 45 nm. The maximum shear stress increases linearly to 10 GPa at the end of the elastic region with *θ* = 76.5° (*γ*_max_ = ~15%). With further torsion, yielding occurs with a sudden drop of *τ*_max_ from 10 GPa to 3.5 GPa. The maximum shear stress shows a jerky behaviour in the entire plastic region (76.5° < *θ* < 225°). The corresponding evolution of mean potential energy as a function of the torsion angle is shown in Figure 1b (black). In the elastic region, the potential energy increases quadratically from −4.062 eV/atom to −4.033 eV/atom. The spectrum of *E*_p_ is jerky also in the plastic region.

Yielding is caused by the nucleation of dislocations. Dislocations first nucleate in the region near the loading ends. We calculated the total length of dislocation lines in the system as a function of torsion angle, as shown in Figure 1c. Dislocations are generated after yielding (*θ* > 76.5°). The total length of the dislocations increases rapidly from 0 to ~400 nm at *θ* = 80°. The relative increase of the dislocation length diminishes for bigger twist angles. The average dislocation length reaches a maximum value at *θ* = 137°, after which the total dislocation length decreased a little and then remains at a constant value of ~500 nm. When normalizing the dislocation length by the volume of the nanowire, we can evaluate the evolution of the dislocation density (right *y* axis in Figure 1c). The maximum dislocation density is about 1.6 × 10^14^ cm^−2^, which is higher than a hugely deformed bulk metal because our torsion deformation is very large (the corresponding *γ*_max_ is about 0.44 at the end of torsion experiment).

The plastic deformation is divided into two regimes based on the dislocation density. In the first regime, the dislocation density increases with increasing torsion angle. In the second regime, the dislocation density is almost unchanged. We can infer that the deformation mechanisms in the first regime is closely related to dislocation avalanches (including nucleation and propagation) but not in the second regime. We define the first regime A as 80° < *θ* < 137° and the second regime B as 137° < *θ* < 225°. The analysis of the regime A does not begin exactly at the yield point, because too many jerks overlap at the very beginning of yielding. It is hard to obtain true statistical behavior for these jerks and we excluded the small region 76.5° < *θ* < 80° from the analysis.

**Figure 2:**
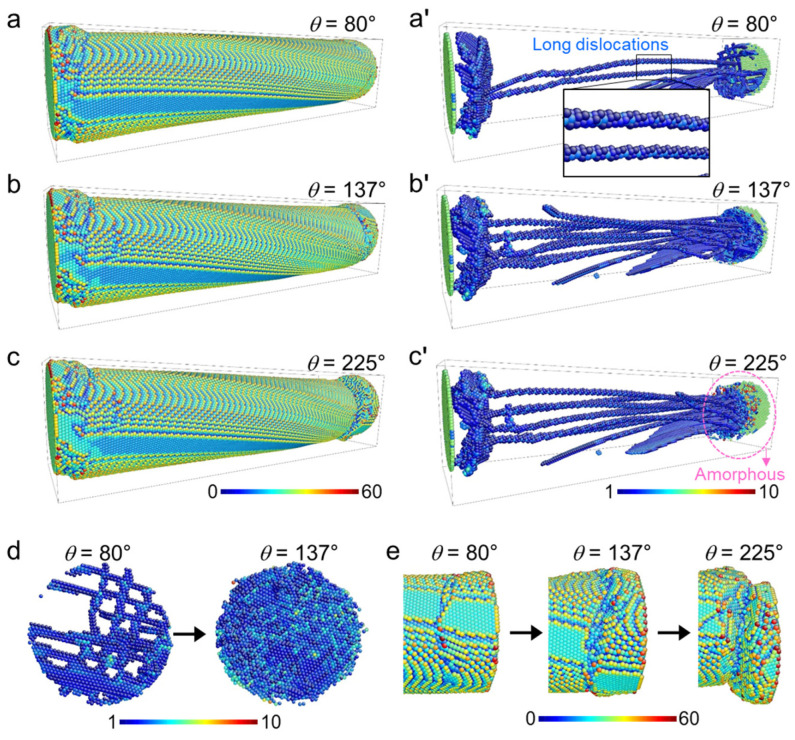
Typical atomic configurations during the torsion deformation of a pristine α-Fe nanowire with diameter of 10 nm and length of 45 nm at 1 K. The atomic configuration at the beginning of region A is shown in (**a**), and the corresponding dislocation structure is shown in (**a’**). The atomic configuration at the beginning of region B (also the end of region A) is shown in (**b**), and the corresponding dislocation structure is shown in (**b’**). The atomic configuration at the end of region B is shown in (**c**), and the corresponding dislocation structure is shown in (**c’**). The evolution of dislocations near the wire ends is shown in (**d**). The increase of the local amorphization near the loading end with increasing twist angle is shown in (**e**). The colors are coded by the centrosymmetry parameter [34].

The plastic deformation in A is dominated by dislocations. Figure 2a,a’ show the atomic configuration and the dislocation patterns at *θ* = 80°. In the near-end region, dislocations are formed by torsion deformation. Some long dislocations extend along the entire nanowire (Figure 1a’). Plastic deformation in A increases the near-end dislocation density (Figure 2d) and generates more long dislocations along the nanowire, as shown in Figure 2b,b’. At the end of regime A (*θ* = 137°), a complex and stable dislocation pattern is formed (Figure 2b,b’). Most dislocations have screw characteristics with Burgers vectors of $\frac{1}{2}$<111> (see the inset of Figure 2a’, Supplementary Materials Figures S1 and S2). The full structural change in regime A can be seen in Supplementary Video S1.

Further deformation in regime B does not cause further increase of the dislocation density. The dislocation patterns are stable in region B, as shown in Figure 2c,c**’**. The decrease of the dislocation density at the beginning of region B is caused by the amorphization in the near-end region (Figure 2c’). The long dislocation patterns are then frozen (see Supplementary Materials Video S2). Almost all plastic deformations in B occur near the loading end (Figure 2e and Supplementary Materials Video S3). No distinct dislocations were found in the near-end region after the deformation in the regime B because stress-induced amorphization replaced the dislocation mechanism.

**Figure 3:**
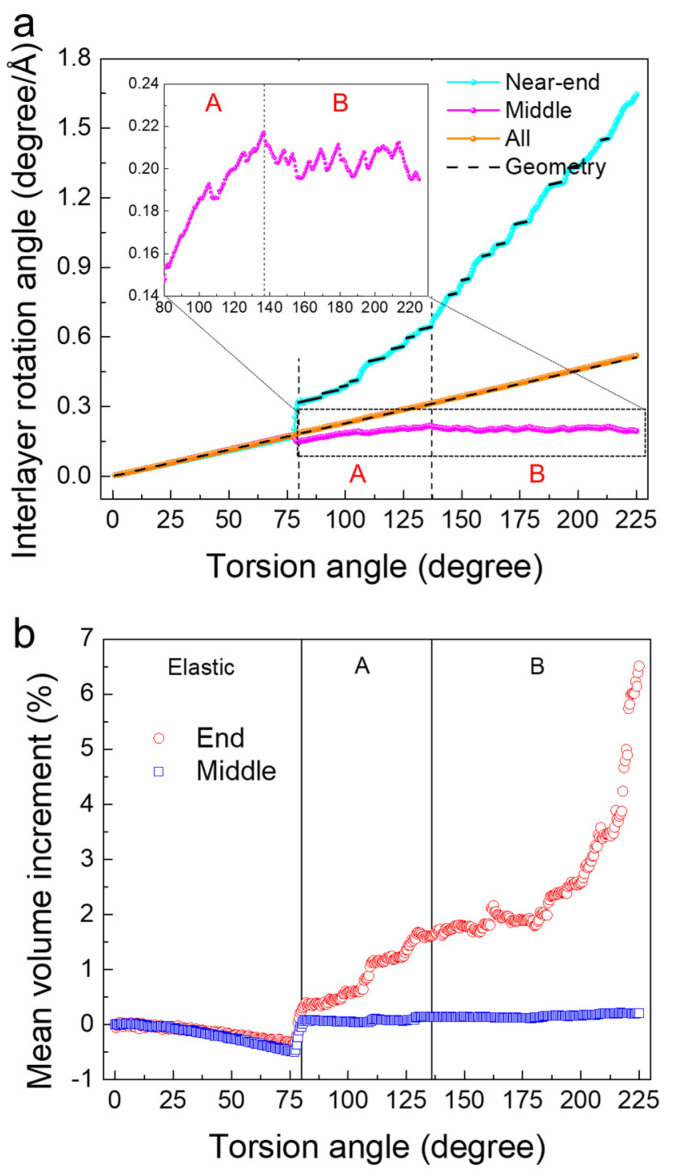
(**a**) Interlayer rotation as functions of torsion angle in the end (cyan), middle (magenta), and the entire sample (orange). The black dashed line indicates the geometrical expectation. (**b**) Mean atomic volume increment as functions of the torsion angle in the end (red) and middle (blue) part.

The interlayer rotation between two adjacent (112) layers in Figure 3 indicates that deformation in region A occurs in both the middle and the end parts of the wire. In contrast, deformation in regime B mainly occurs near the loading ends. We calculated the interlayer rotation between two adjacent (112) layers and normalized them by the layer distance. Then we averaged them over the corresponding regions (near-end in cyan and middle parts in magenta in Figure 3a). The average value over the entire sample (orange) was the same as the geometrical expectation, which was the ratio of the total rotation angle to the total length of the nanowire. In the elastic stage, the interlayer rotation in the middle (magenta) and in the end (cyan) and the geometrical expectation were the same. After yielding, the relative rotation at the ends was much larger than in the middle, which stemmed from the high defect and dislocation density at the end parts. The long dislocations in the middle release the elastic deformation of the matrix induced lower relative rotations than the geometrical values. The increase of the rotation angle in regime A indicated that the plastic deformation occurred mainly in the middle part. The rotation saturated in region B (see the inset in Figure 3a), when the applied tilt was absorbed at the end parts.

Plastic deformation in the near-end region was mainly caused by dislocations that form at the beginning of regime A. With increasing twist angle, the regions with a high density of dislocations began to ‘melt’. There were no dislocations recognized near the end in regime B, indicating an obvious amorphization. Further deformation induced stronger “melting” near the end parts. Large blocks (or grains) were seen to swim in the melt (see Supplementary Materials Video S4).

The melting-like behavior near the loading end in region B induced a volume increment, as shown in Figure 3b (red curve). In comparison, the volume increment in the middle part remained constant (blue). The total volume increment in regime B near the loading end was ~5%, which was comparable with the melting induced volume expansion in metals [35]. In region A, the volume increment near the end (between −0.32% and 1.64%) was much larger than in the middle (between −0.5% and 0.14%). This coincided with the high dislocation density and a melting-like behavior in very small regions near the end (further information in Supplementary Materials Figure S3).

### 3.2. Mixed Wild Plasticity Induced by Dislocation Avalanches

The jerk strength *J* is defined as *J*~(d*E*_p_/d*t*)^2^, where d*t* is a timestep (5 fs in our statistical analysis). The jerk strength is correlated with the energy fluctuations, indicating some discontinuous structural evolution (e.g., dislocation nucleation, dislocation motion etc.) inside the sample. We now undertake a statistical analysis of *J* to better understand the dynamics of torsion plasticity of pristine α-Fe nanowires. Generally speaking, a power-law PDF indicates a collaborative or wild behavior, while an exponential PDF indicates a mild or random dynamic behavior [20].

**Figure 4:**
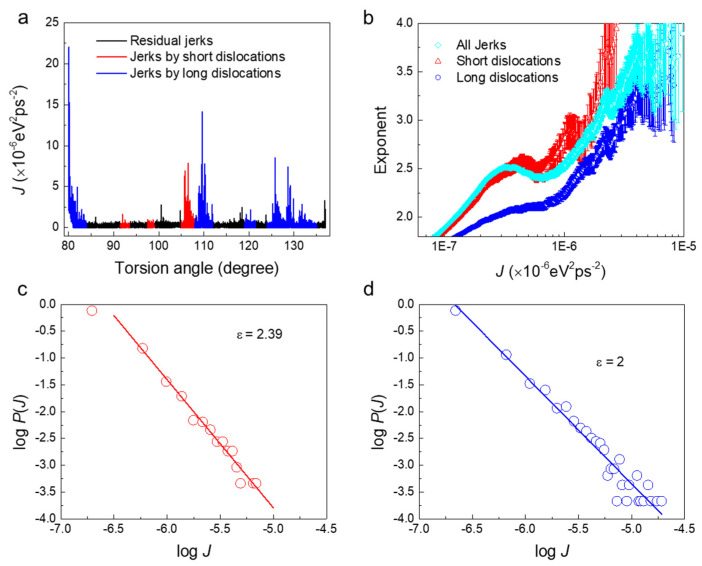
Statistical analysis of the jerk strength for pristine nanowires under twisting at stage A. (**a**) Jerk spectrum at 80° < *θ* < 137°. The jerks stem from movements of short dislocations near the loading end (red) and from long dislocations along the length direction (blue). (**b**) Exponents found by the maximum likelihood (ML) analysis of all jerks (cyan), jerks induced by short dislocations (red) and jerks induced by long dislocations (blue). (**c**) Log-log plot of the probability distribution function (PDF) of jerks induced by short dislocations (red in Figure 4a) showing a power-law distribution with an exponent of 2.39. (**d**) PDF log-log plot of jerks induced by long dislocations (blue in Figure 4a) showing a power-law distribution with an exponent *ε* = 2.0.

The jerk strength spectrum evolves as a function of the torsion angle in A (80° < *θ* < 137°) as shown in Figure 4a. The jerky spectrum indicates that the growth and movement of dislocations are not smooth processes. The PDF of jerk strengths exhibits a mixed power-law distribution, *P*(*J*)~*J*^−*ε*^, which is also validated by the maximum likelihood (ML) method (magenta line in Figure 4b). The mixed power-law indicates two different deformation mechanisms in region A.

**Figure 5:**
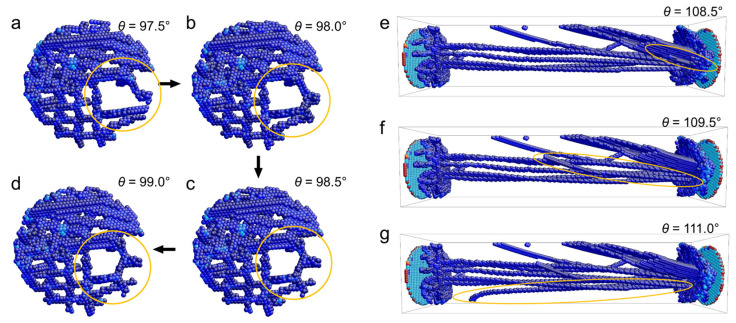
Typical atomic configurations during the evolution of (**a**–**d**) short dislocations near the loading end and (**e**–**g**) long dislocations along the wire. Typical dislocation segments are encircles in orange.

The motion of dislocations along the radial direction is strongly limited by the sample size and interactions with other dislocations while the motion along the axial direction is less of a constraint. We now describe some typical processes in A, namely the atomic-scale evolution of short dislocations near the end sections (Figure 5a–d) and long dislocations along the wire length (Figure 5e–g). We then distinguish between the jerks due to the movement of short dislocations perpendicular to the wire length (red in Figure 4a) and long dislocations along the wire length (blue in Figure 4a). The jerks induced by short dislocations (red) follow a power-law distribution with an exponent of 2.39 (Figure 4c) and those induced by long dislocations (blue) follow a power-law distribution with an exponent of 2.0 (Figure 4d). The exponents evaluated by the ML method in Figure 4b confirm this result.

Figure 5 shows the motion of short and long dislocations in the nanowire under torsion. Before the movement, a typical dislocation pattern has been formed (Figure 5a). Only very short dislocation segments begin to move (orange circles from Figure 5a–d). The induced jerks are very weak, therefore. The long dislocation movements are very different. Figure 5e–g show the appearance and growth of two long dislocations, indicated by orange circles. These movements induce much bigger jerks. Note that we distinguish all jerks by checking the corresponding atomic configurations. Here, only some ‘typical’ structure changes are captured. When the structure evolution is too complex to be recognized in detail or when the movement of short and long dislocations are mixed together, the jerks are not analyzed further (residual black jerks in Figure 4a). Nevertheless, distributions of jerk strength induced by short and long dislocations in twisting nanowire are shown to be different, which is the origin of the mixed power-laws in regime A.

**Figure 6:**
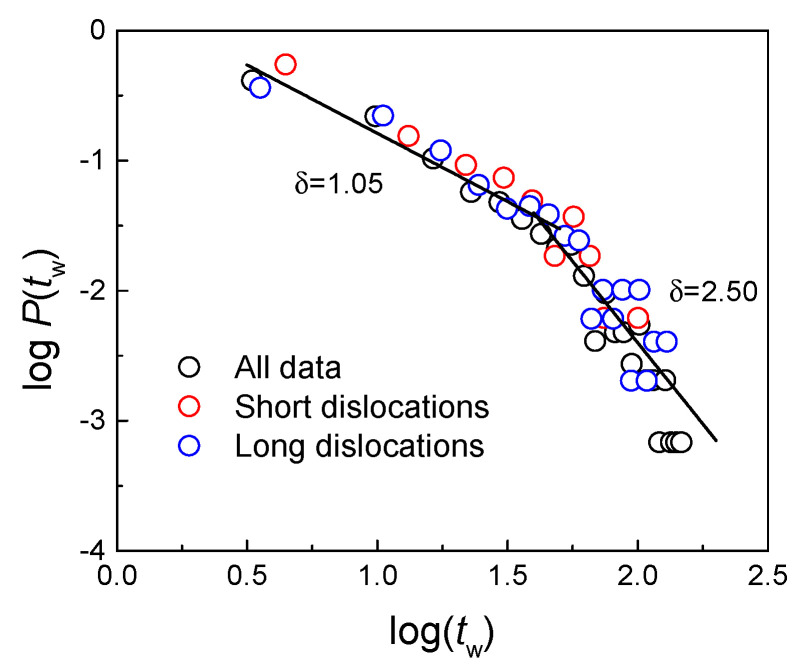
Double power-law distribution of waiting times between successive jerks induced by short dislocation (red), long dislocations (blue) and all (black).

The power-law distributed PDF implies that the plasticity in regime A is highly correlated and “wild” [20]. Wild jerks are correlated in time and Figure 6 shows the PDF of waiting times between successive jerks. Here the waiting time is defined by a jerk strength threshold, which equals 0.01 of the maximum jerks in this period. The PDF of waiting times shows a two-stage power-law distribution with a higher exponent of 1.05 and lower exponent of 2.50. The ratio *R* between the mean value and the standard deviation of waiting times in A is 0.509 (<1), indicating an intermittent waiting time distribution [9]. The PDF of waiting times between jerks induced by short and long dislocation movements are shown separately (red and blue circles in Figure 6), and they follow the same power laws as already seen other studies [36,37].

### 3.3. Mild Plasticity Induced by Local Melting

**Figure 7:**
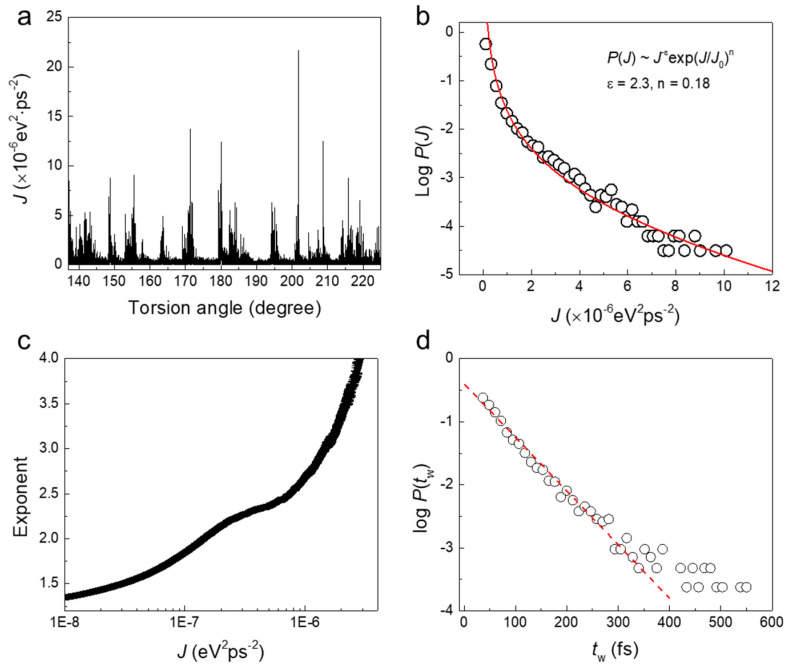
Statistical analysis of jerk strength for a twisted nanowire in the regime B. (**a**) Jerk spectrum at 137° < *θ* < 225°. (**b**) Semi-log plot of the PDF of jerk strengths showing a generalized Poisson distribution. The stretching exponent is *n* = 0.18. (**c**) Evaluated exponent by the ML method, where there is a small plateau at *ε* = ~2.3. The ML curve shows highly damped dynamics. (**d**) Exponential distribution of waiting times between successive jerks.

The jerk spectrum in regime B (137° < *θ* < 225°) is highly non-stationary, as shown in Figure 7a. The PDF shows complex jerk behavior which contains a mixing between power-law and exponential jerk statistics, as shown in Figure 7b. The PDF of jerk strength in regime B can be described by a stretched exponential (generalized Poisson) distribution as *P*(*J*)~*J*^−ε^⋅exp(*J*/*J*_0_)*^n^* [38]. The stretching exponent *n* equals 0.18 and *ε* equals 2.3, which agree well with the evaluated exponent by ML method in Figure 7c, which shows a small plateau at *ε* = ~2.3. The ML curve also shows a high degree of damping [39].

The large twist angle at regime B leads to local amorphization or “melting” of the parts of the wire close to the clamped end-layers. Further torsion does not induce further twists in the rest of the sample, and the long dislocation pattern is stable (Figure 2b,c and Supplementary Materials Video S2). The dislocation movements lead to avalanche behavior with power-law distributed energy jerks. The ‘melting’ process breaks the coherency of the structure. The distribution of the PDF of jerk strength *J* in Figure 7b is roughly exponential over two decades.

The local “melting” in region B also changes the waiting time distribution of jerks. The PDF of waiting times between successive jerks follows an exponential distribution (Figure 7d) in contrast with the power law distributions in Figure 6 for the long dislocations. The ratio *R* between the mean value and the standard deviation of waiting times at this stage equals 1.07, indicating the deformation in B is ‘mild’ [11,20].

## 4. Discussion

We analyzed the plastic deformation mechanisms and the dynamic behavior of pristine α-Fe nanowires under torsion. The torsion plasticity for nanowires without defects is dominated by dislocations for small twist angles. These dislocations transverse the length of the sample and release the torsion generated by the twist of the clamped layers at either end of the wire. High twist angles, at the crossover between regime A and regime B, lead to amorphization, or “melting”, of parts of the wire close to the clamped end-layers. There is no further torsion in the rest of the sample (region B) and the dislocations do not change. The dislocation movements in the middle of the sample lead to power-law distributed avalanches. The ‘melting’ process breaks the coherence of the structure and shows complex jerking behavior which contains some exponential jerk statistics. Their jerk statistics is akin to stick-slip models as observed at rough interfaces and in Earthquakes [40,41,42].

**Figure 8:**
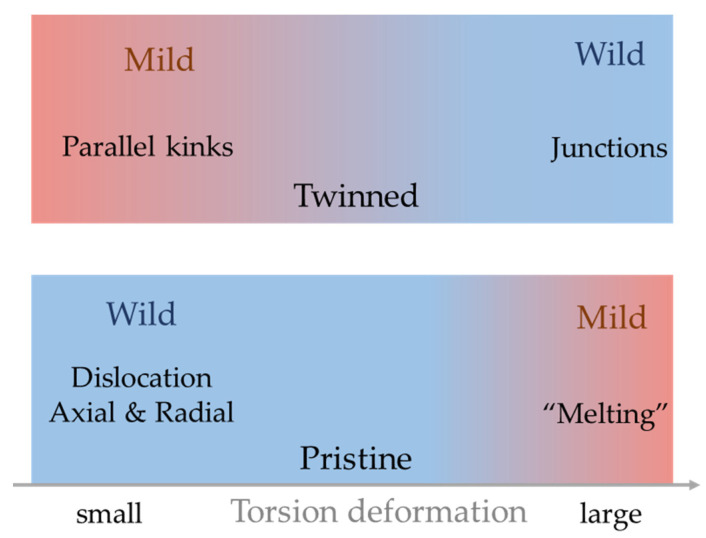
Comparison the deformation dynamics of twinned and pristine α-Fe nanowires under torsion.

The deformation mechanisms and the jerk dynamics of our pristine twisting α-Fe nanowires (Figure 8) are very different from those in twinned [11] nanowires of the same material. In twinned samples, a two-stage deformation process was found [11]. The plastic deformation first proceeds by parallel kinks formation in individual twin walls. The motion of such kinks generates uncorrelated jerks and is associated with mild avalanches. Further deformation induces the interaction between kinks, forming kink junctions. The plasticity is now dominated by the pinning–depinning processes of junctions and generates wild correlated jerks.

The dynamics of the dislocation-dominated plasticity can be tuned by many factors, such as the sample size, the defects distribution, the dislocation entanglements etc. With the decrease of sample size, the dislocation motion behaves with “smaller is wilder” behavior [21]. The interaction between dislocations and defects, e.g., clusters or precipitates, and the interaction between dislocations are both related to the locking of the dislocation patterns. A stable dislocation pattern leads to mild plasticity [20,21]. In thin nanowires, dislocations nucleate near the loading end, and grow fast along the axial direction. The nucleation and fast growth processes proceed by ‘wild’ avalanches. Dislocation movement along the axial direction and radial direction follows a different power-law exponent. Long dislocations are pinned near the end of the sample. Therefore, when the dislocation density saturates, the dislocation pattern is frozen. Further deformation leads to local amorphization or melting, which generates mild plasticity. Dislocation dominated plasticity is not only observed in α-Fe metallic nanowires, but also experimentally observed in other metallic nanowires such as Cu, Ag, Au etc. [43,44,45]. The dynamic behavior of dislocation induced plasticity is, therefore, expected to be similar in different materials due to the same one-dimensional geometry. However, twinning and dislocations often occur together. The dynamic behavior of dislocations, which are affected by the twin boundaries [46], leads to more complex behaviour. However, this phenomenon goes beyond the scope of our present study. Further investigations will be undertaken to tackle this question.

## Data Availability

The data presented in this study are available on request from the corresponding authors.

## Supplementary Materials

The following are available online at https://www.mdpi.com/article/10.3390/nano11061602/s1, Figure S1: Dislocation structure characteristic analysis at *θ* = 137°, Figure S2: Atomic dislocation-core structure at *θ* = 137°, Figure S3: Atomic volume increment at *θ* = 76.5°, Video S1: Dislocation pattern evolution in regime A, Video S2: Stable dislocation pattern in regime B, Video S3: Local melting near the end in regime B, Video S4: Large blocks (or grains) swim in the melt.
