# Supplementary material for: Twisting of a Pristine α-Fe Nanowire: From Wild Dislocation Avalanches to Mild Local Amorphization
Source: Nanomaterials (Basel). 2021 Jun 18;11(6):1602. doi: 10.3390/nano11061602 (PMC8234800; doi:10.3390/nano11061602)
Supplement: Supplementary file 1 [file nanomaterials-11-01602-s001.zip › nanomaterials-1252693 sup final.pdf]

# Twisting of a Pristine α-Fe Nanowire: From Wild Dislocation Avalanches to Mild Local Amorphization

**Yang Yang [1], Xiangdong Ding [1,*], Jun Sun [1] and Ekhard K. H. Salje [1,2,*]**

[1] State Key Laboratory for Mechanical Behaviour of Materials, School of Materials Science and Engineering, Xi'an Jiaotong University, Xi'an 710049, China; yangymse@xjtu.edu.cn (Y.Y.); junsun@mail.xjtu.edu.cn (J.S.)
[2] Department of Earth Sciences, University of Cambridge, Cambridge CB2 3EQ, UK
* Correspondence: dingxd@mail.xjtu.edu.cn (X.D.); ekhard@esc.cam.ac.uk (E.K.H.S.)

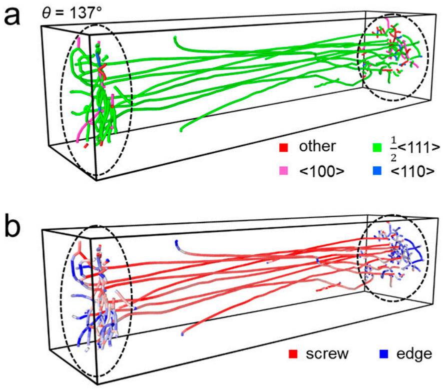


Figure S1. Dislocation structure characteristic analysis at $\theta = 137°$. (a). Burgers vectors and (b) dislocation types are analyzed by the Dislocation Extraction Algorithm (DXA) in the structural visualization package OVITO [28, 29]. The dashed circles indicate the dislocation wall near the loading end.

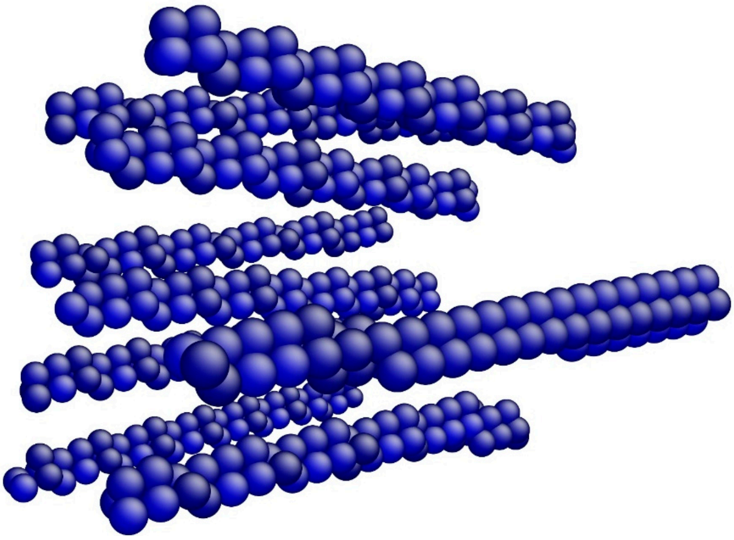

Figure S2. Atomic dislocation-core structure at $\theta = 137°$.

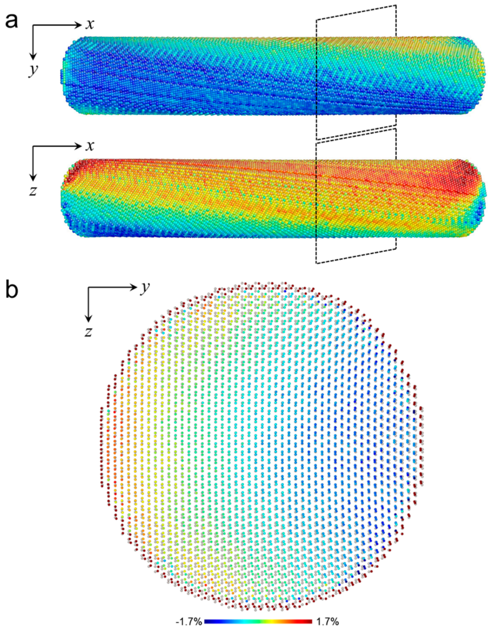


Figure S3. Atomic volume increment at $\theta = 76.5°$. The grey atoms are at $\theta = 0°$ and the color atoms are at $\theta = 76°$. They are colored by the voronoi volume change.
